# Supplementary material for: Social support correlates with glucocorticoid concentrations in wild African elephant orphans
Source: Commun Biol. 2022 Jul 14;5:630. doi: 10.1038/s42003-022-03574-8 (PMC9283395; doi:10.1038/s42003-022-03574-8)
Supplement: Supplementary file 2 — Supplementary Information [file 42003_2022_3574_MOESM2_ESM.pdf]

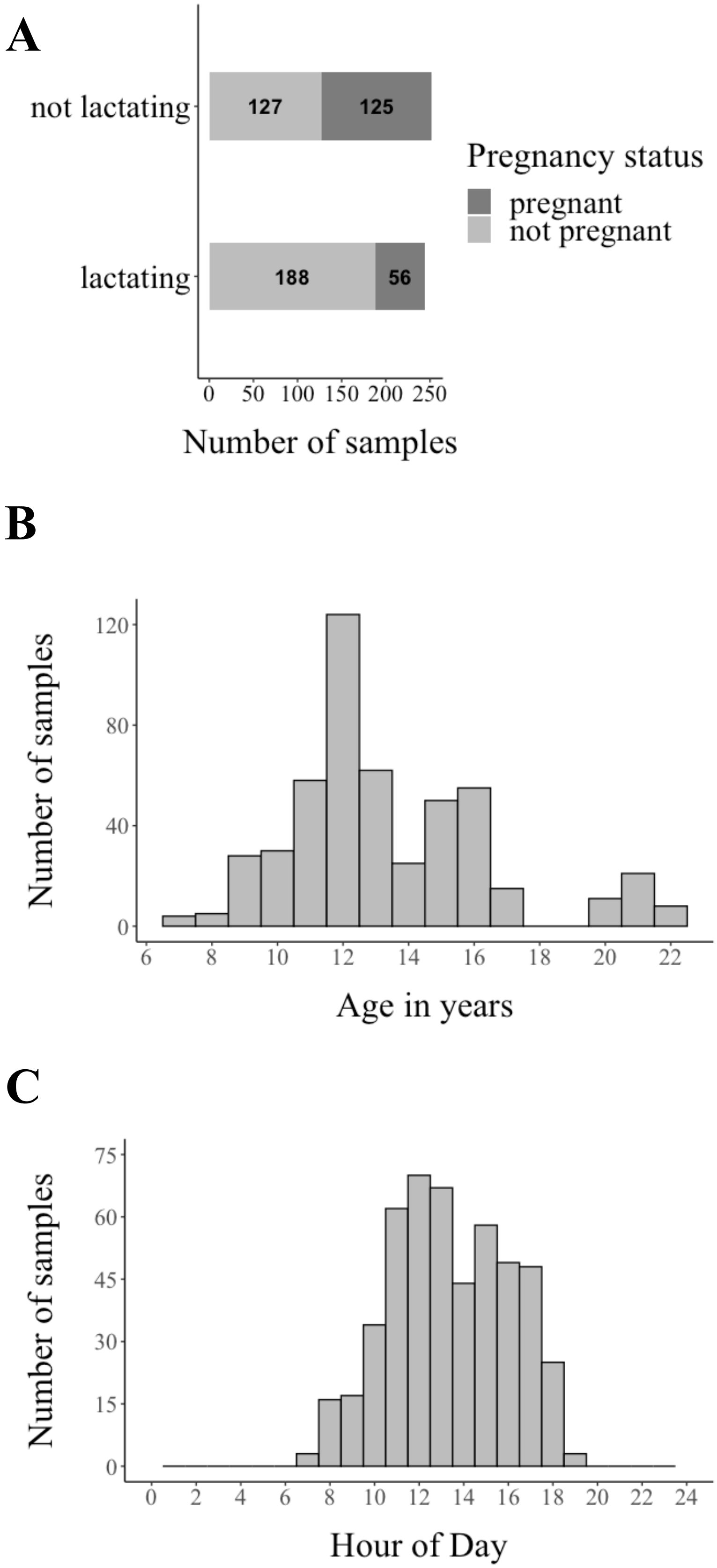
A
not lactating
lactating
127
125
188
56
Pregnancy status
pregnant
not pregnant
0 50 100 150 200 250
Number of samples
B
Number of samples
0 40 80 120
6 8 10 12 14 16 18 20 22
Age in years
C
Number of samples
0 15 30 45 60 75
0 2 4 6 8 10 12 14 16 18 20 22 24
Hour of Day

D

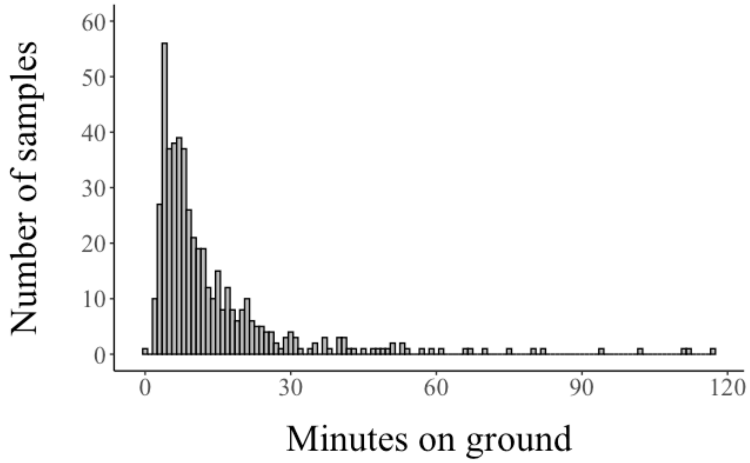

Number of samples
0
10
20
30
40
50
60
0
30
60
90
120
Minutes on ground

E

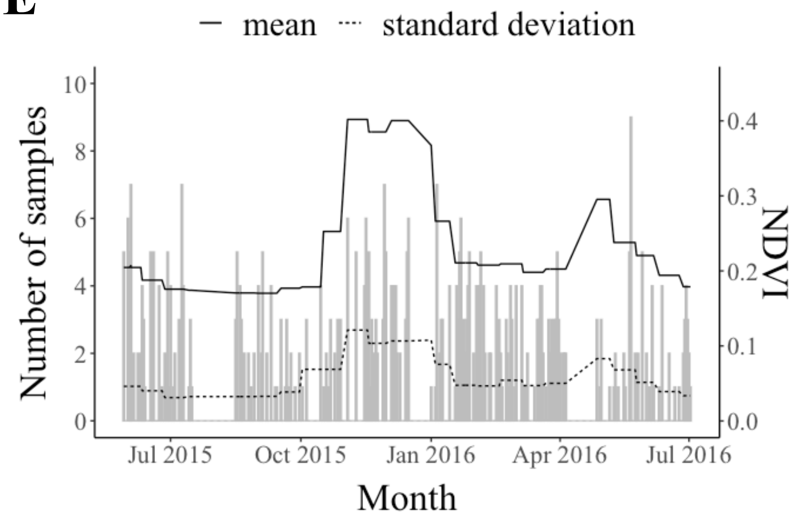

mean
standard deviation
Number of samples
0
2
4
6
8
10
NDVI
0.0
0.1
0.2
0.3
0.4
Jul 2015
Oct 2015
Jan 2016
Apr 2016
Jul 2016
Month

F

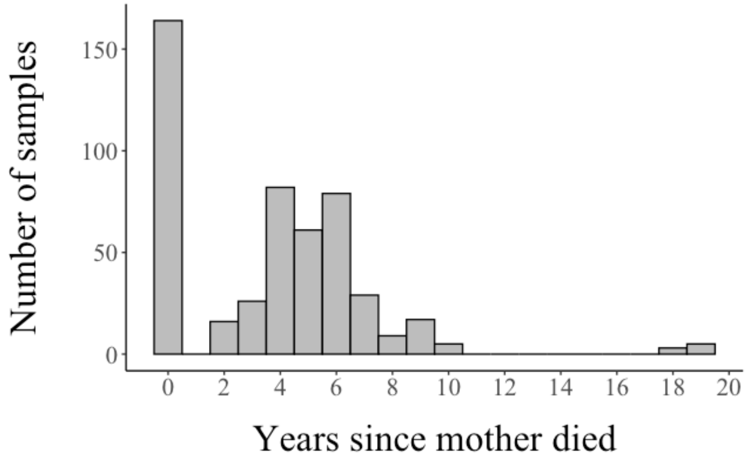

Number of samples
0
50
100
150
0
2
4
6
8
10
12
14
16
18
20
Years since mother died

G

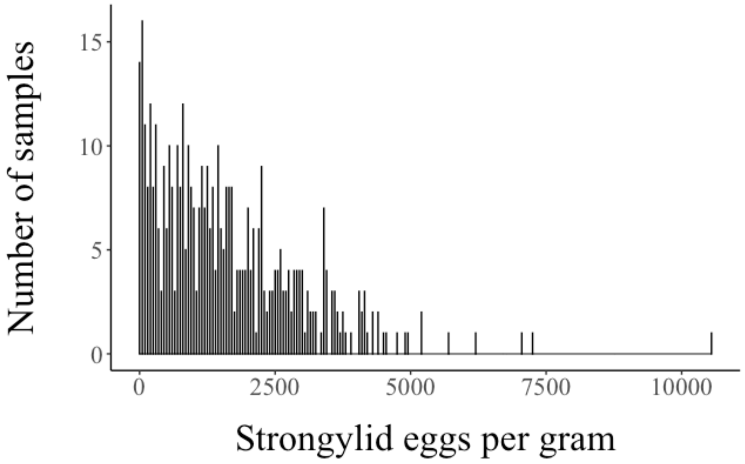

Supplementary Figure 1: Time-variant covariates. A) Bar chart showing number of samples collected from females who were lactating versus not, colored according to whether they were collected from pregnant versus not pregnant females. B-G) Histogram of samples according to age of the female they were collected from, time of day they were collected, amount of time spent on the ground prior to collection, month of collection (including lines showing corresponding mean and standard deviation of NDVI according to the secondary y-axis), years since mother died (non-orphan samples were assigned a value of zero), and estimated number of strongylid eggs per gram of fecal matter from the same sampling event. NDVI stands for “normalized difference vegetation index,” a measure of primary productivity.

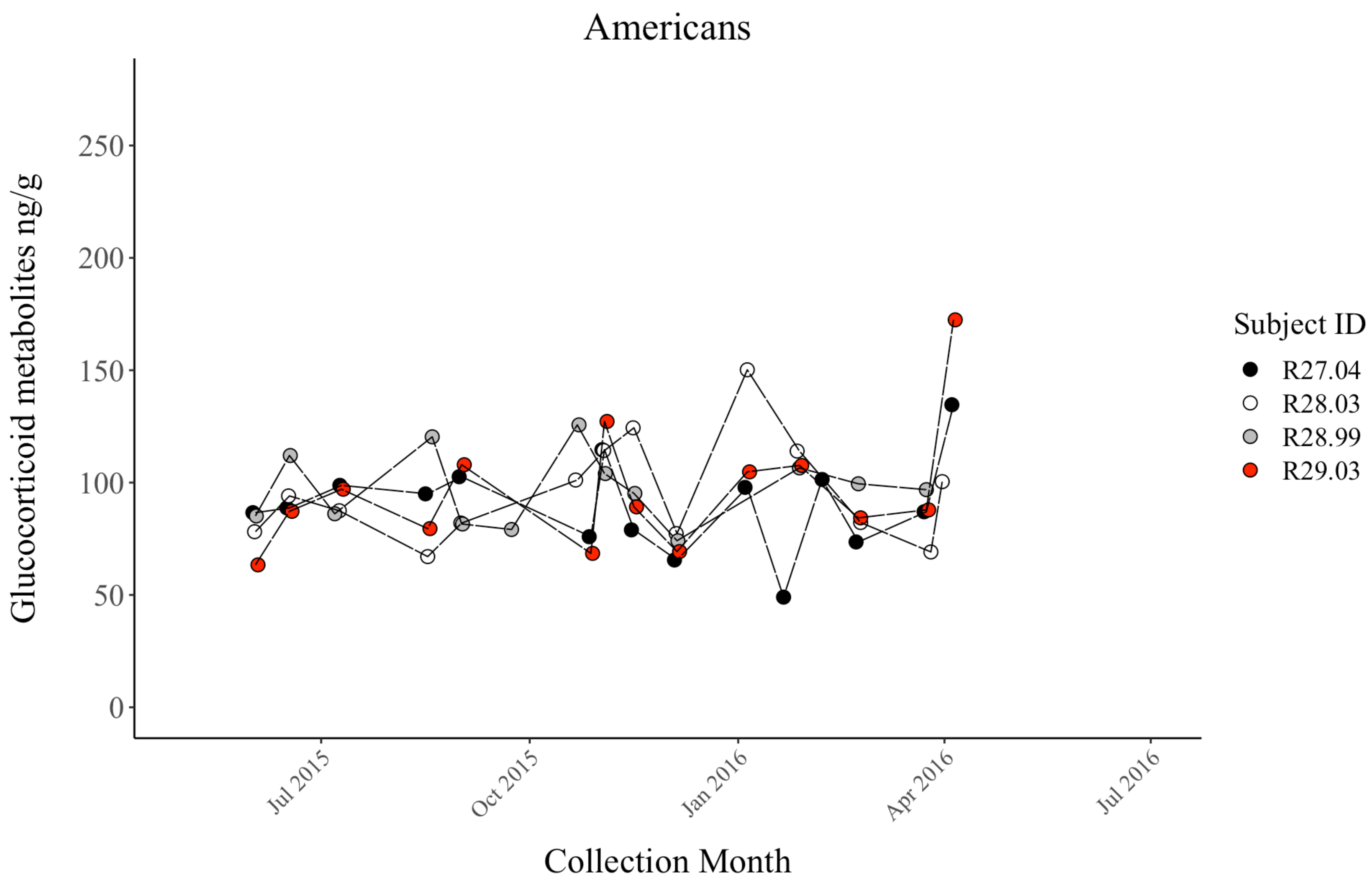

Americans
Glucocorticoid metabolites ng/g
0
50
100
150
200
250
Jul 2015
Oct 2015
Jan 2016
Apr 2016
Jul 2016
Collection Month
Subject ID
R27.04
R28.03
R28.99
R29.03

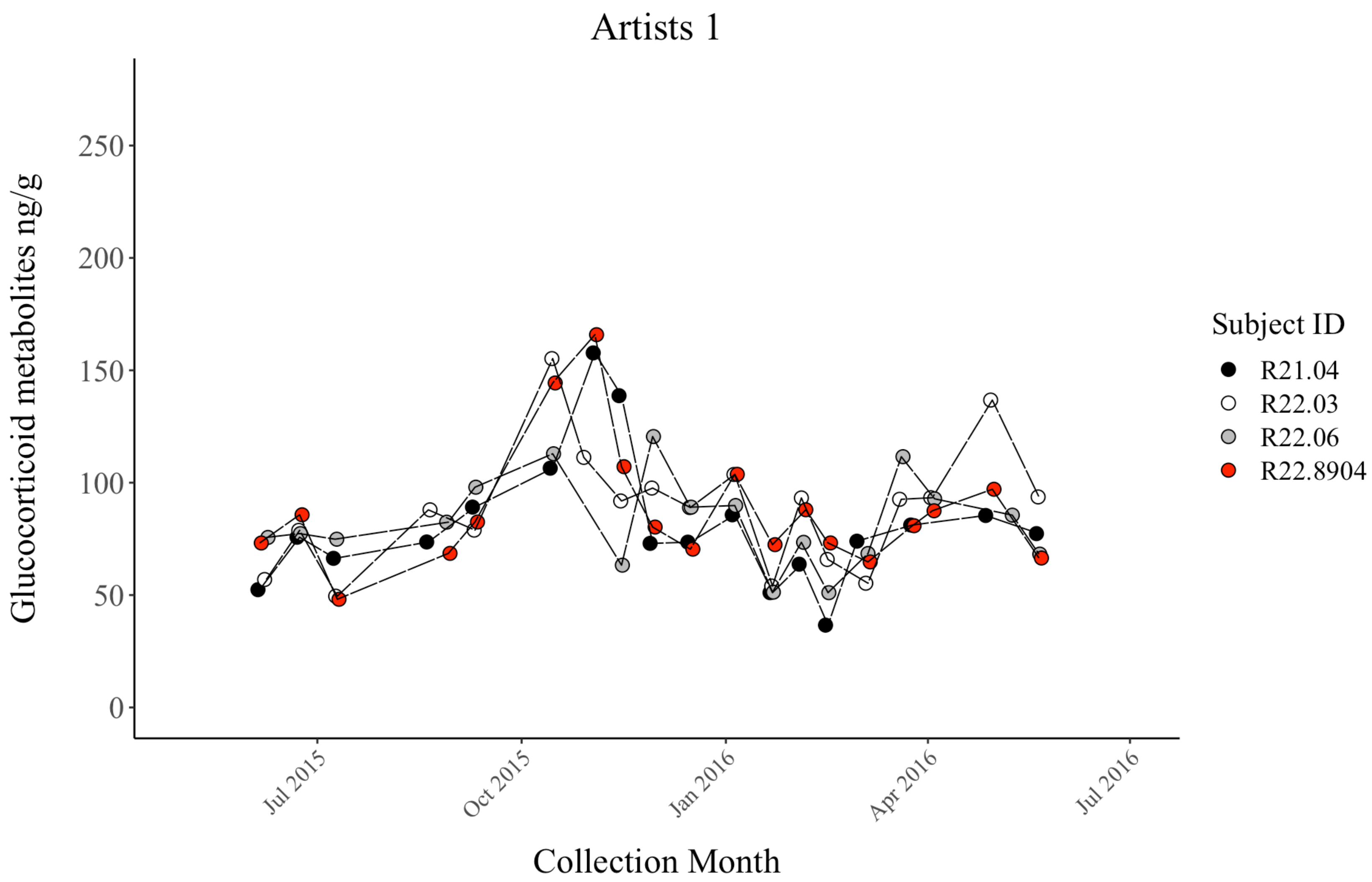

Artists 1
Glucocorticoid metabolites ng/g
250
200
150
100
50
0
Jul 2015
Oct 2015
Jan 2016
Apr 2016
Jul 2016
Collection Month
Subject ID
R21.04
R22.03
R22.06
R22.8904

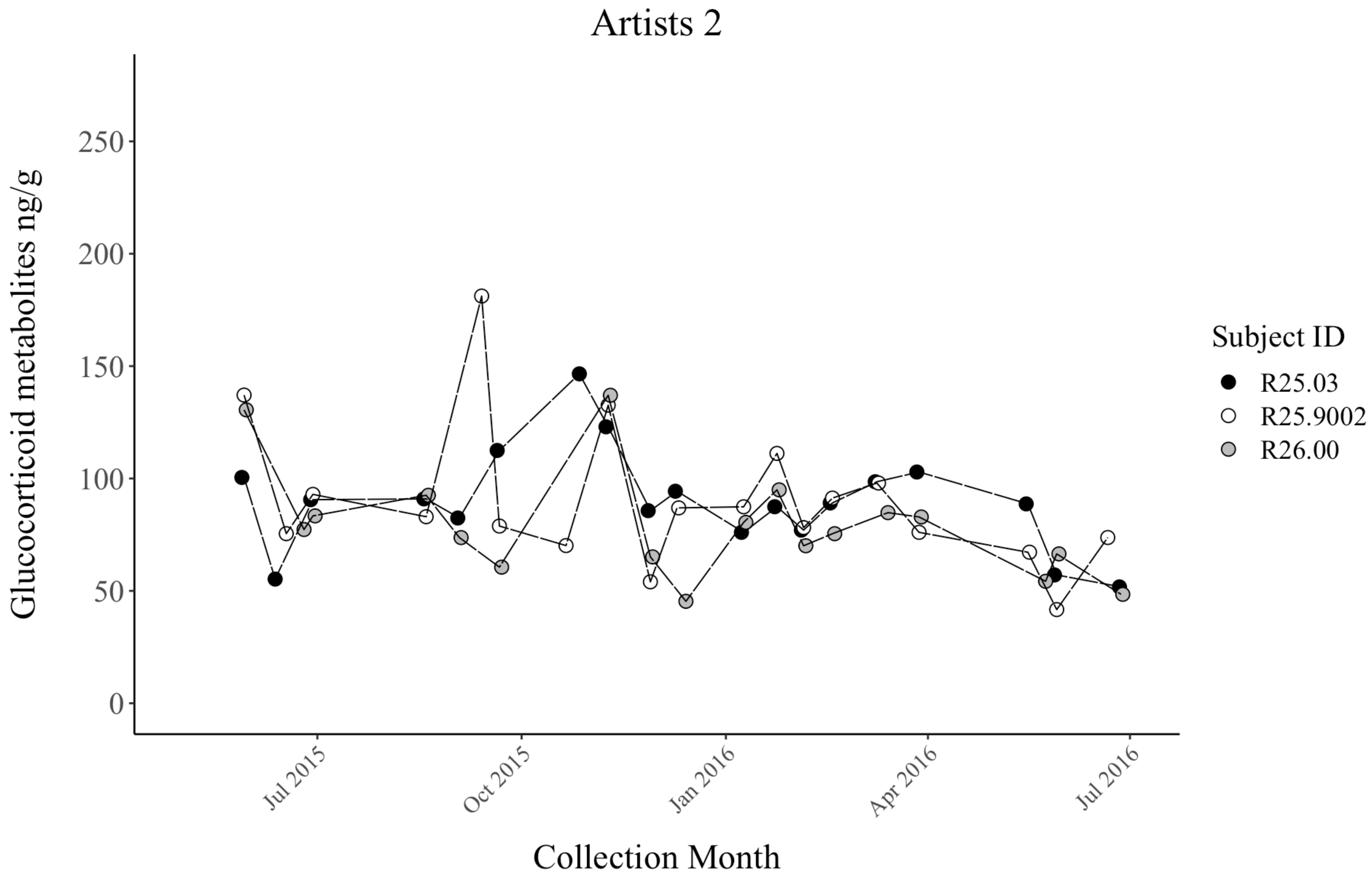

Artists 2
Glucocorticoid metabolites ng/g
250
200
150
100
50
0
Jul 2015
Oct 2015
Jan 2016
Apr 2016
Jul 2016
Collection Month
Subject ID
R25.03
R25.9002
R26.00

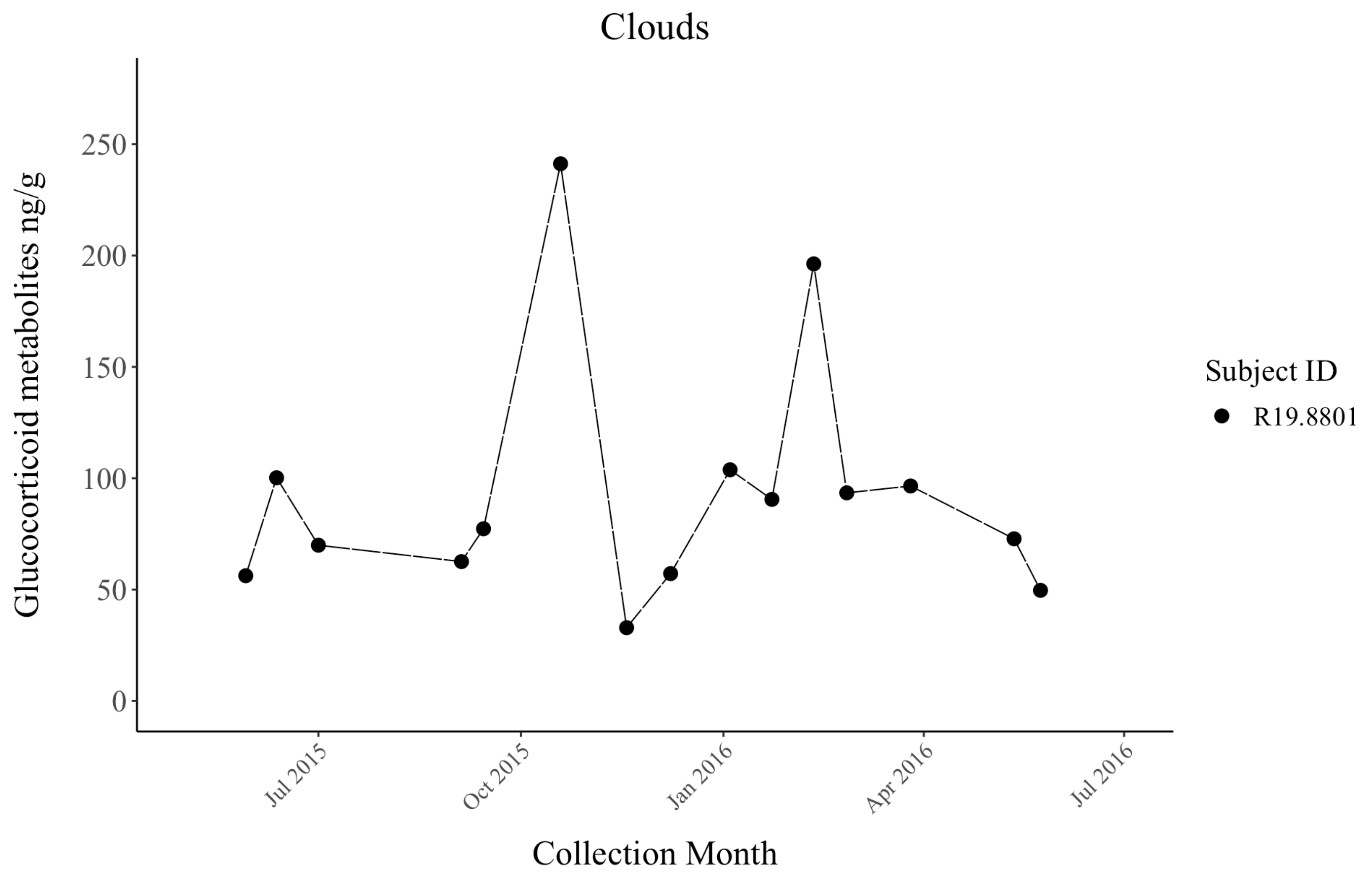
Clouds
Glucocorticoid metabolites ng/g
250
200
150
100
50
0
Jul 2015
Oct 2015
Jan 2016
Apr 2016
Jul 2016
Collection Month
Subject ID
R19.8801

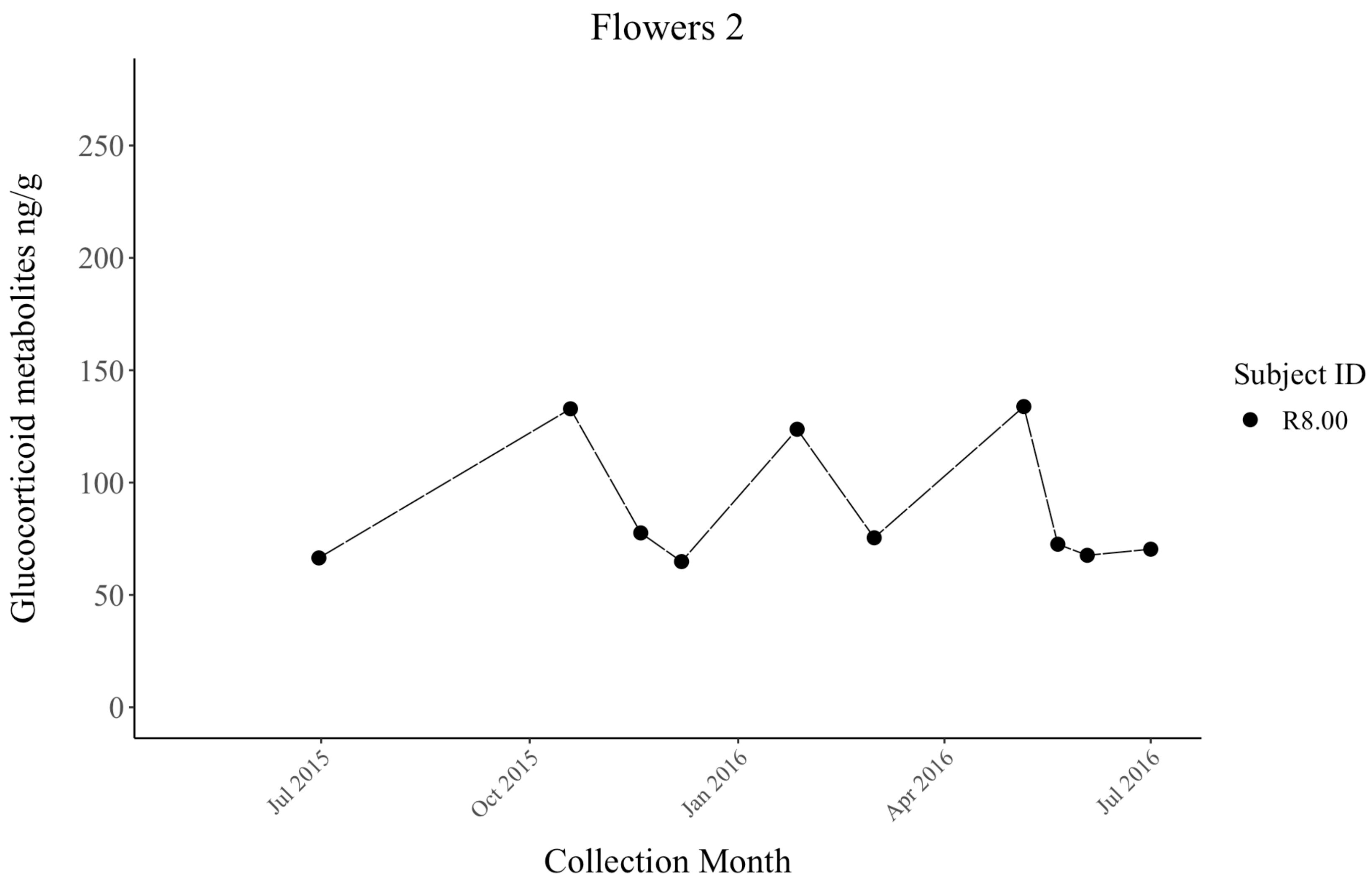
Flowers 2
Glucocorticoid metabolites ng/g
0
50
100
150
200
250
Jul 2015
Oct 2015
Jan 2016
Apr 2016
Jul 2016
Collection Month
Subject ID
R8.00

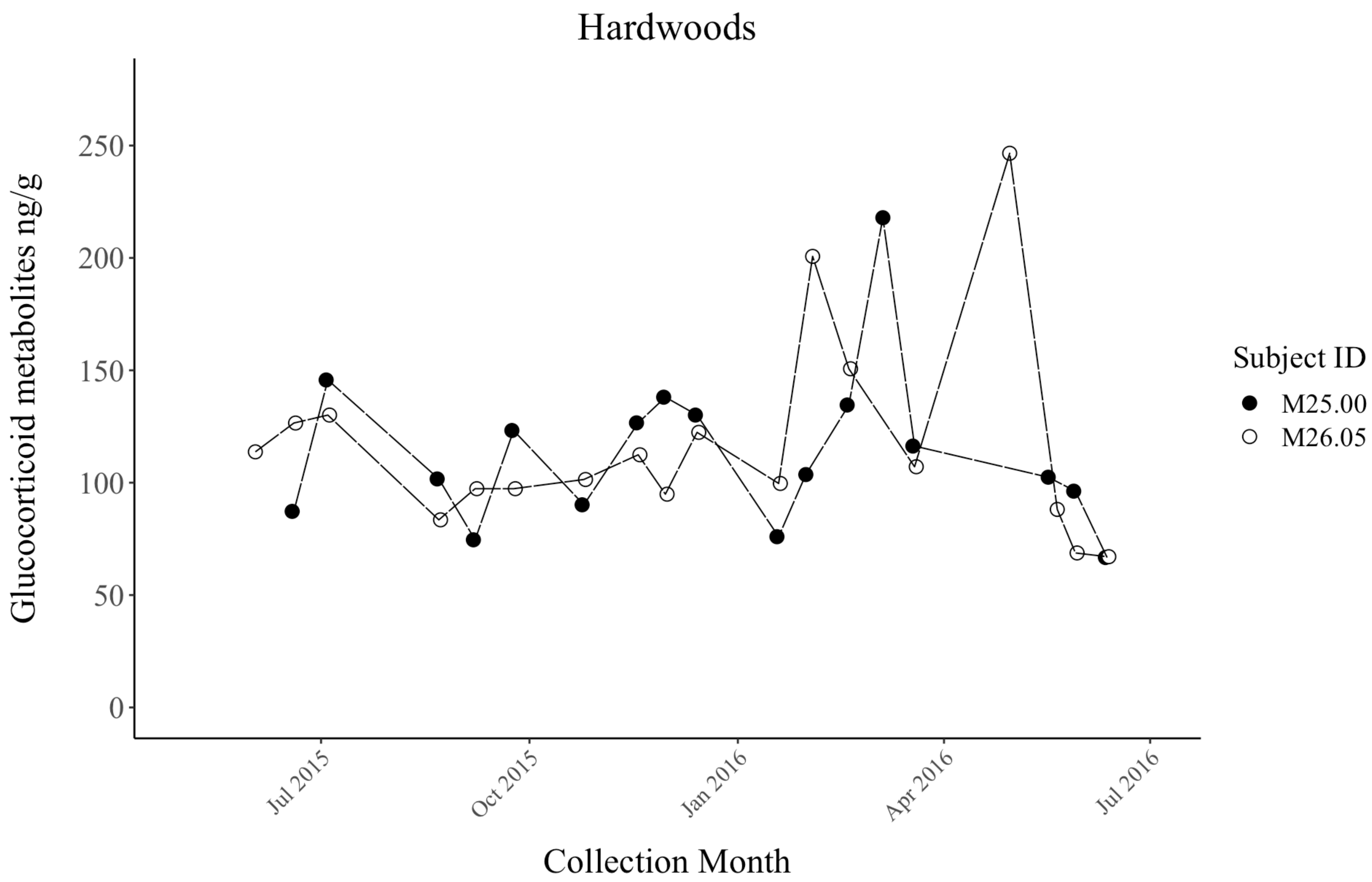
Hardwoods
Glucocorticoid metabolites ng/g
0
50
100
150
200
250
Jul 2015
Oct 2015
Jan 2016
Apr 2016
Jul 2016
Collection Month
Subject ID
M25.00
M26.05

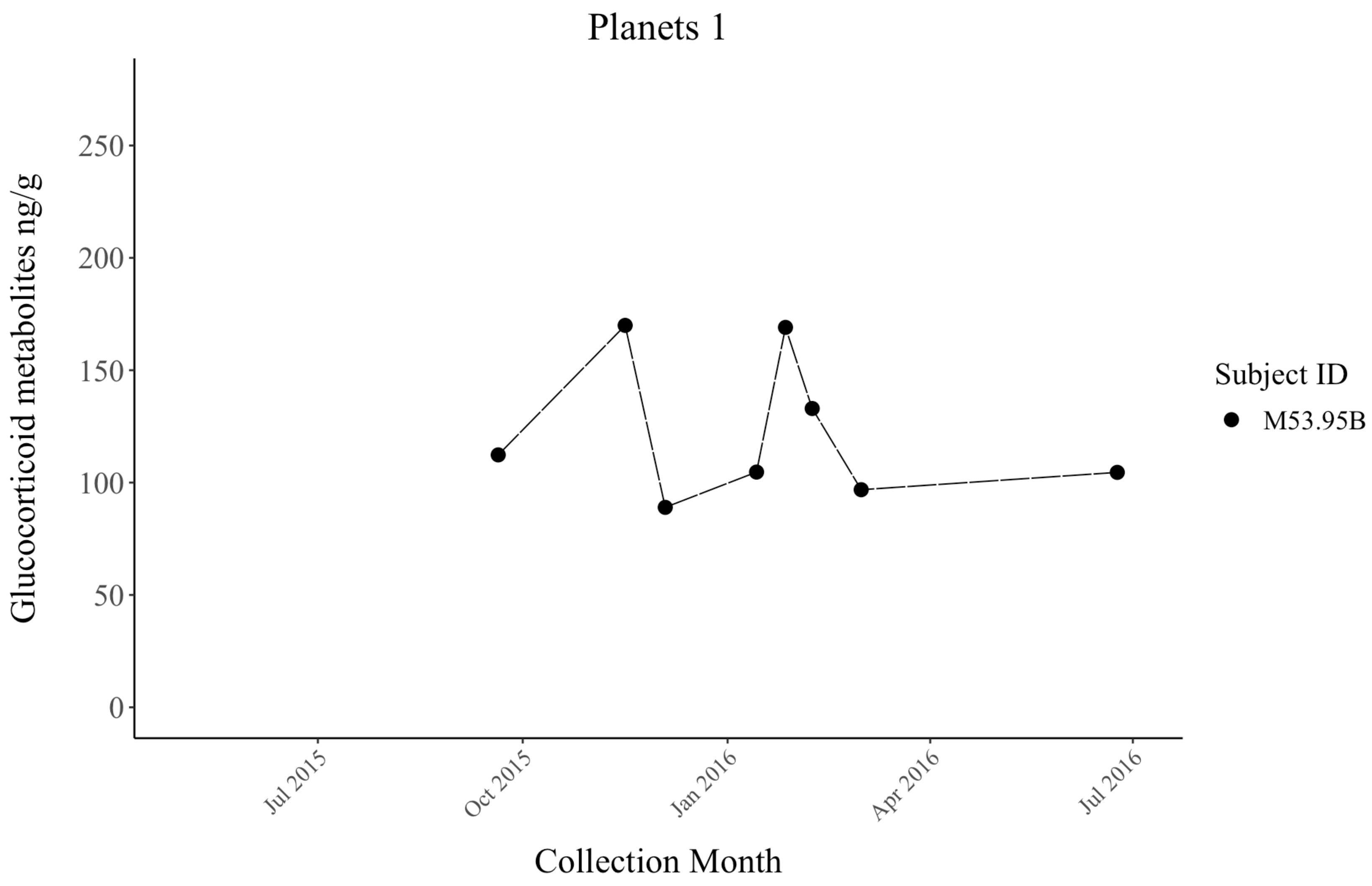

Planets 1
Glucocorticoid metabolites ng/g
250
200
150
100
50
0
Jul 2015
Oct 2015
Jan 2016
Apr 2016
Jul 2016
Collection Month
Subject ID
M53.95B

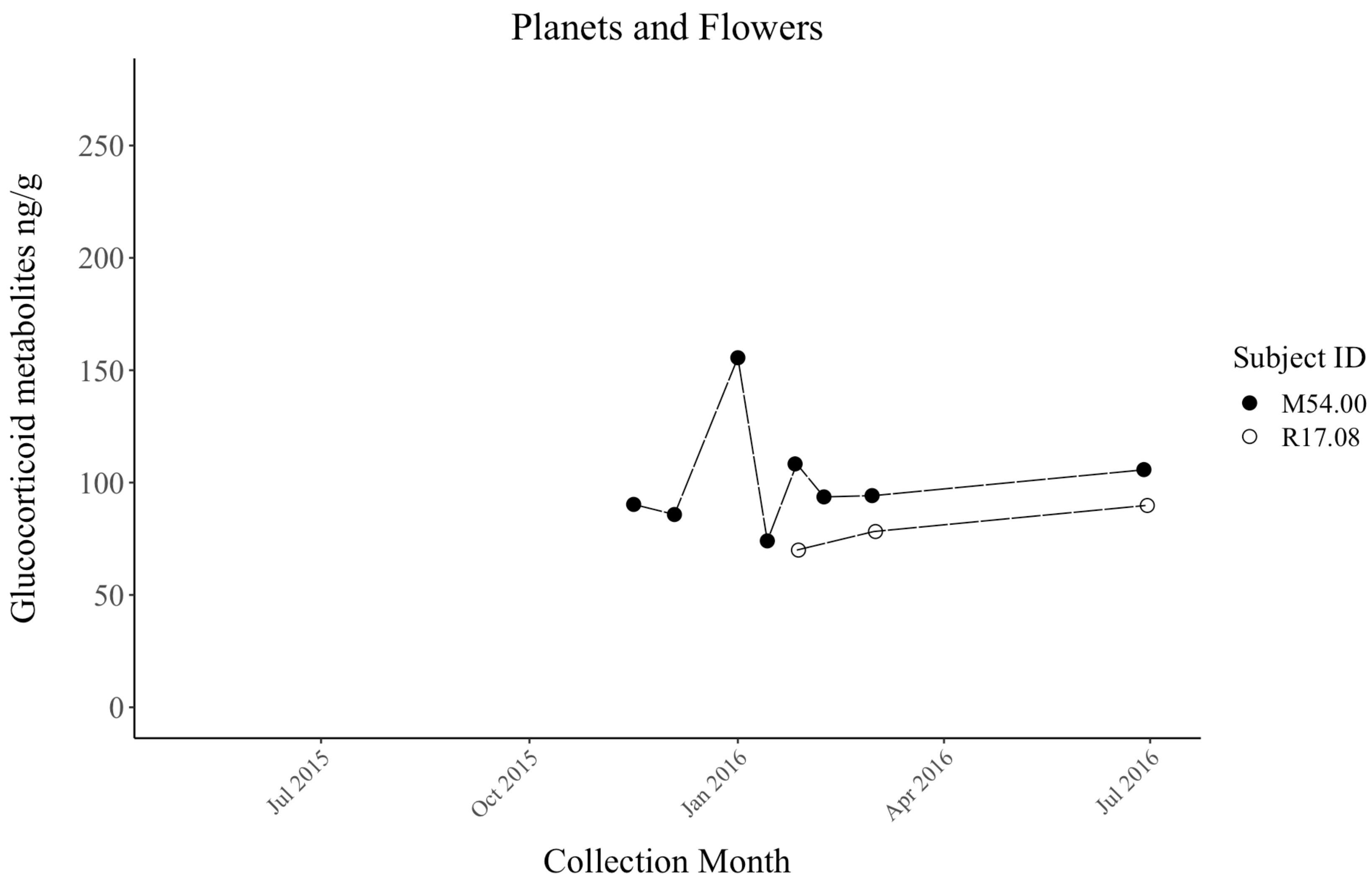

Planets and Flowers
Glucocorticoid metabolites ng/g
250
200
150
100
50
0
Jul 2015
Oct 2015
Jan 2016
Apr 2016
Jul 2016
Collection Month
Subject ID
M54.00
R17.08

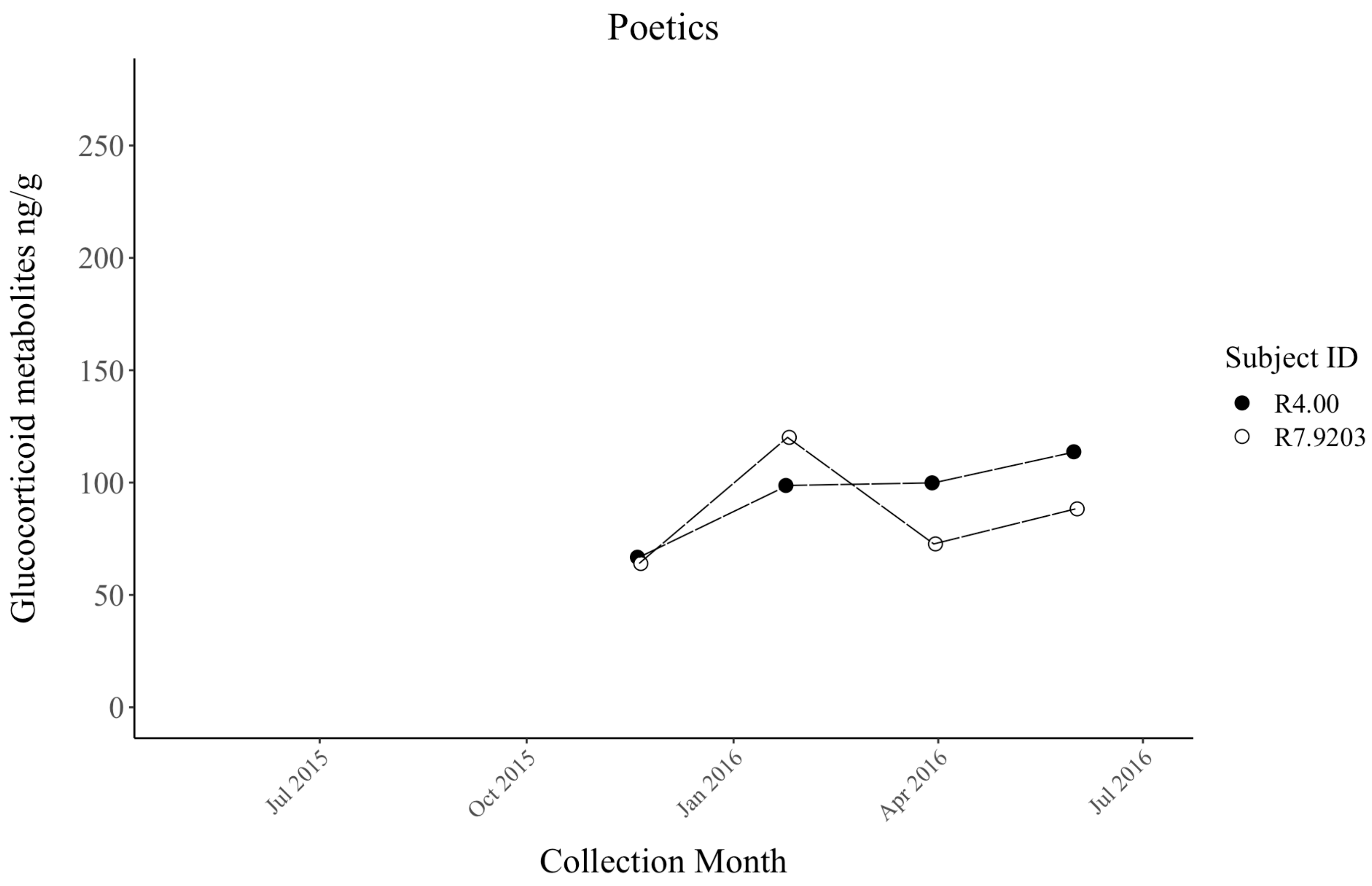

Poetics
Glucocorticoid metabolites ng/g
0
50
100
150
200
250
Jul 2015
Oct 2015
Jan 2016
Apr 2016
Jul 2016
Collection Month
Subject ID
R4.00
R7.9203

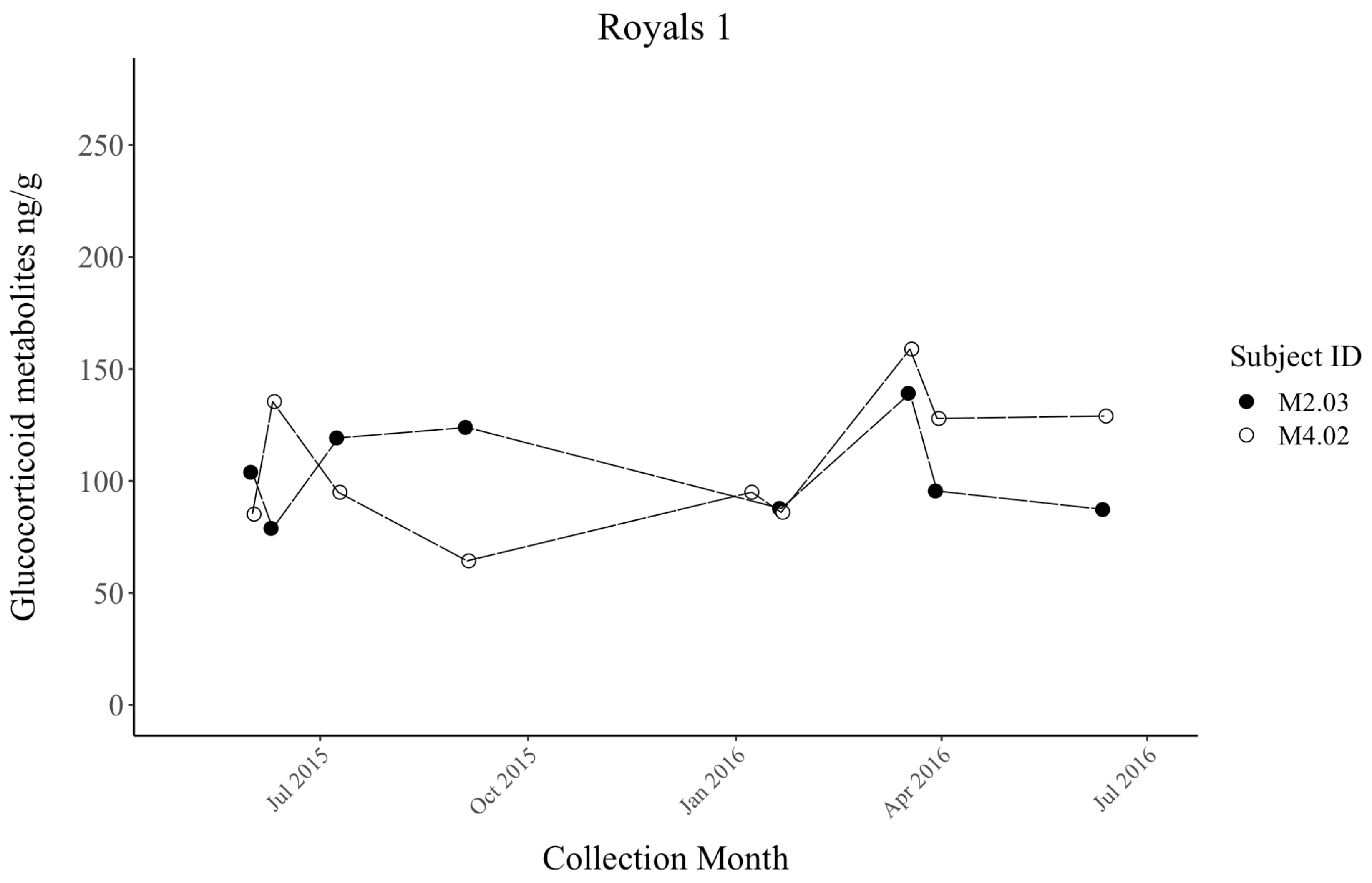

Royals 1
Glucocorticoid metabolites ng/g
0
50
100
150
200
250
Jul 2015
Oct 2015
Jan 2016
Apr 2016
Jul 2016
Collection Month
Subject ID
M2.03
M4.02

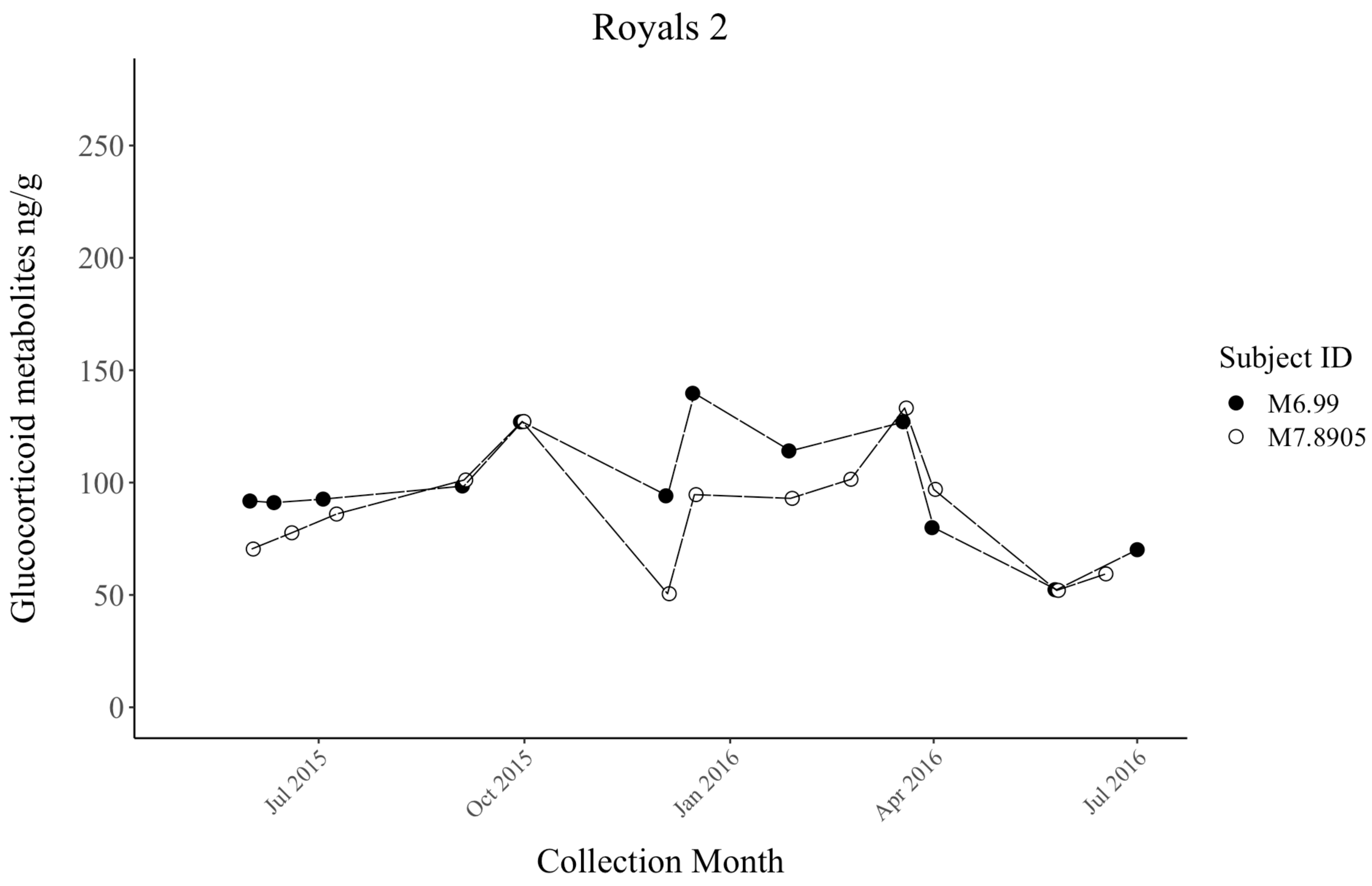

Royals 2
Glucocorticoid metabolites ng/g
250
200
150
100
50
0
Jul 2015
Oct 2015
Jan 2016
Apr 2016
Jul 2016
Collection Month
Subject ID
M6.99
M7.8905

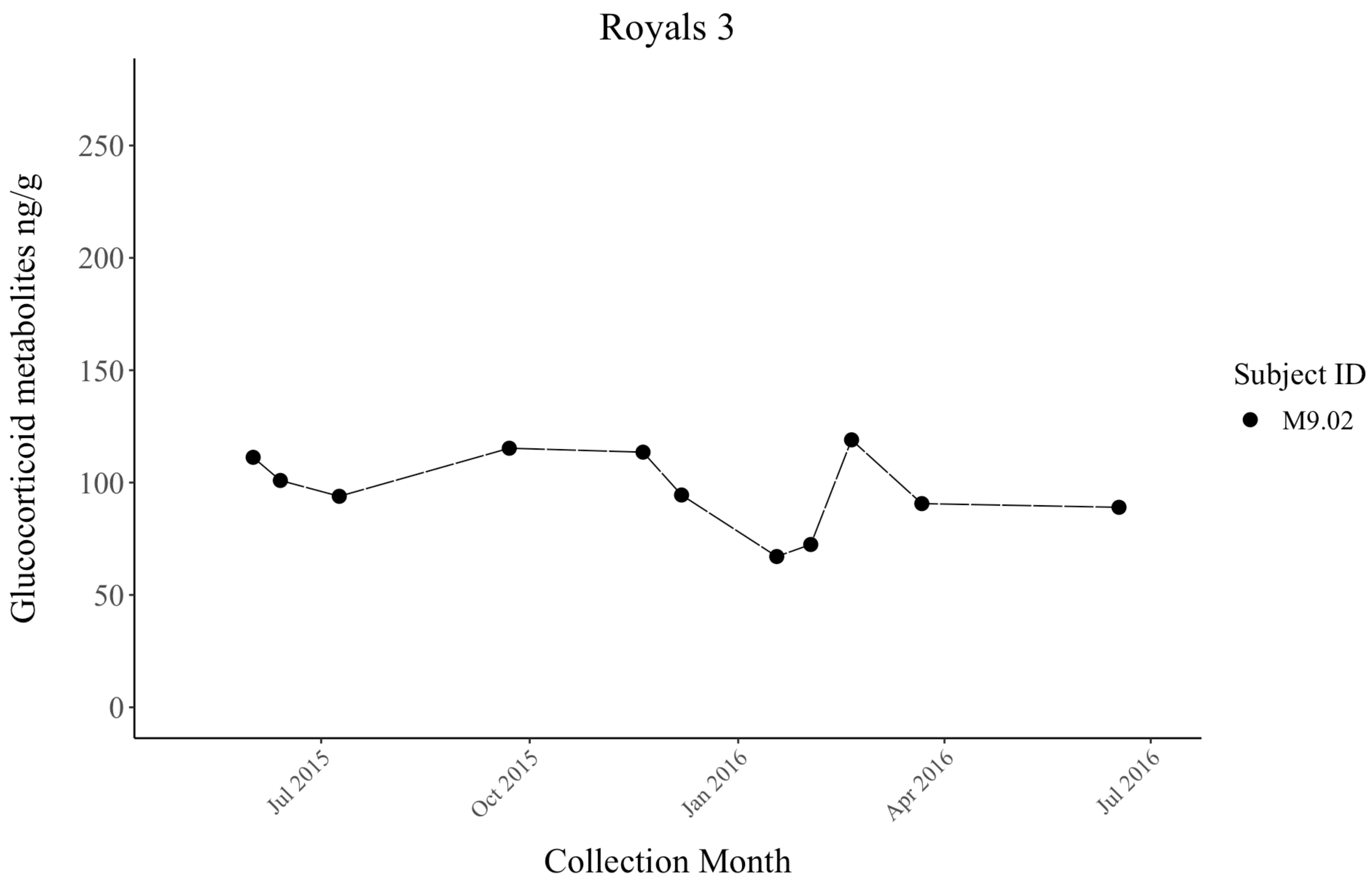
Royals 3
Glucocorticoid metabolites ng/g
250
200
150
100
50
0
Jul 2015
Oct 2015
Jan 2016
Apr 2016
Jul 2016
Collection Month
Subject ID
M9.02

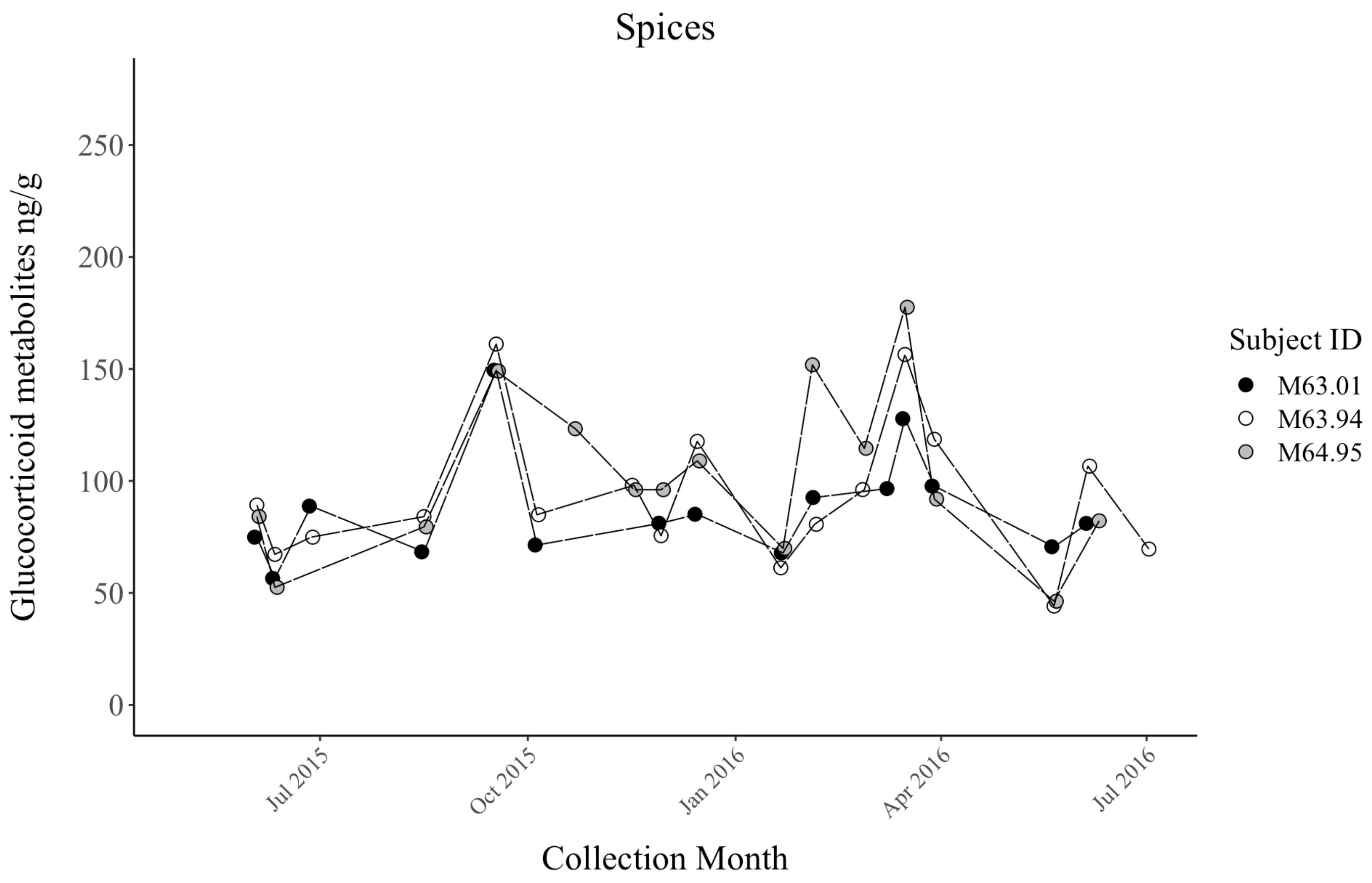

Spices
Glucocorticoid metabolites ng/g
250
200
150
100
50
0
Jul 2015
Oct 2015
Jan 2016
Apr 2016
Jul 2016
Collection Month
Subject ID
M63.01
M63.94
M64.95

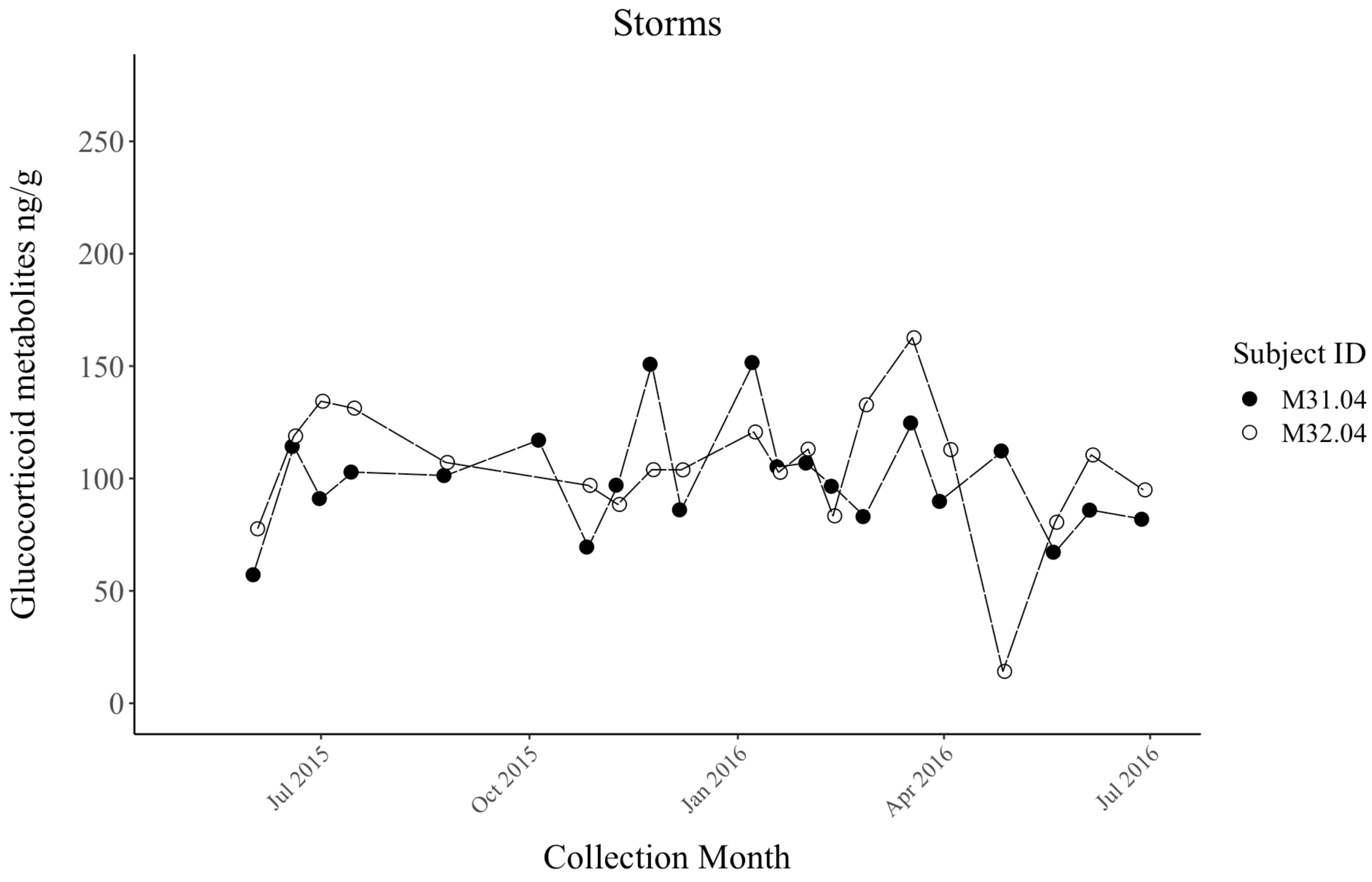
Storms
Glucocorticoid metabolites ng/g
250
200
150
100
50
0
Jul 2015
Oct 2015
Jan 2016
Apr 2016
Jul 2016
Collection Month
Subject ID
M31.04
M32.04

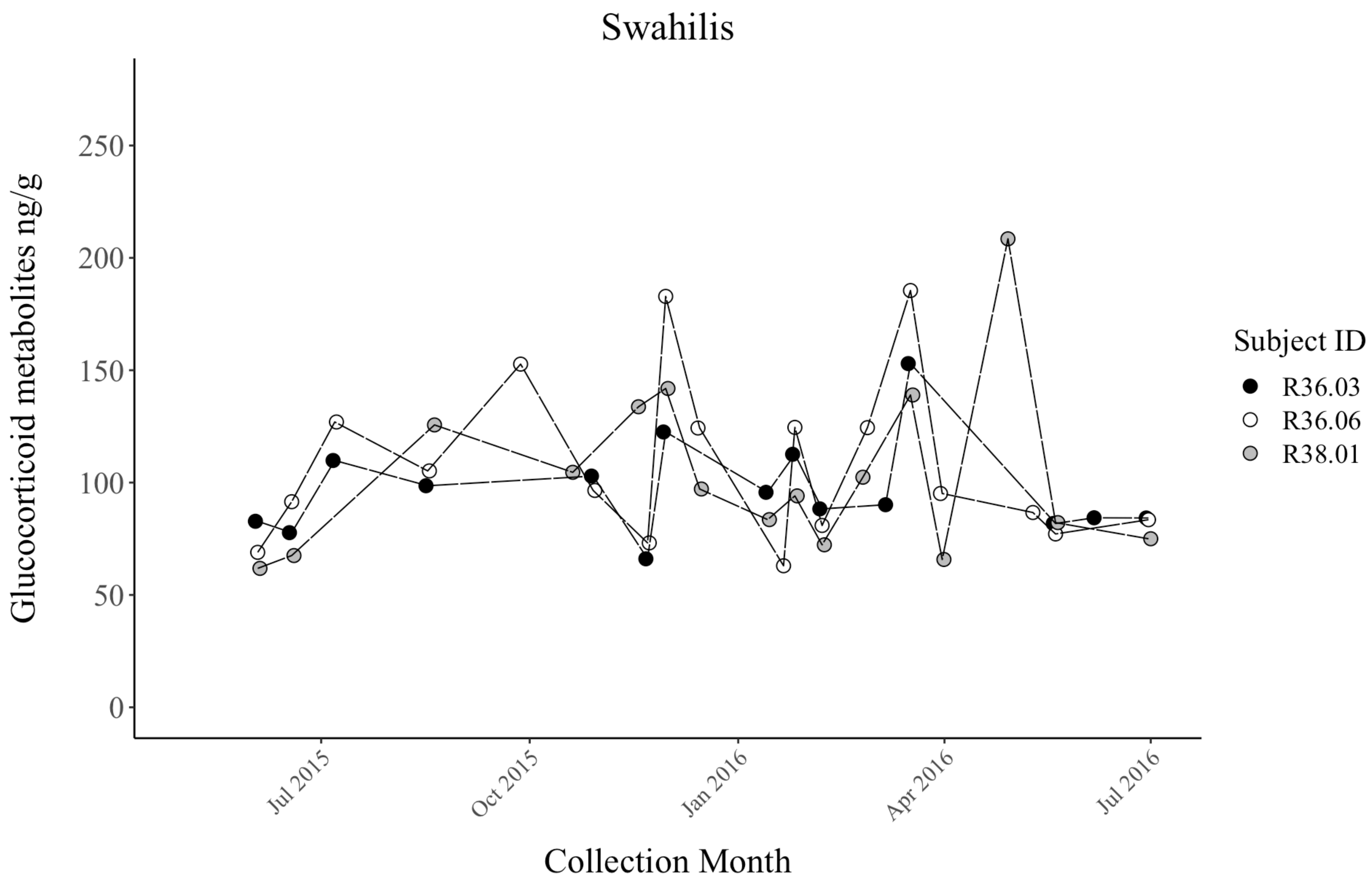

Swahilis
Glucocorticoid metabolites ng/g
0
50
100
150
200
250
Jul 2015
Oct 2015
Jan 2016
Apr 2016
Jul 2016
Collection Month
Subject ID
R36.03
R36.06
R38.01

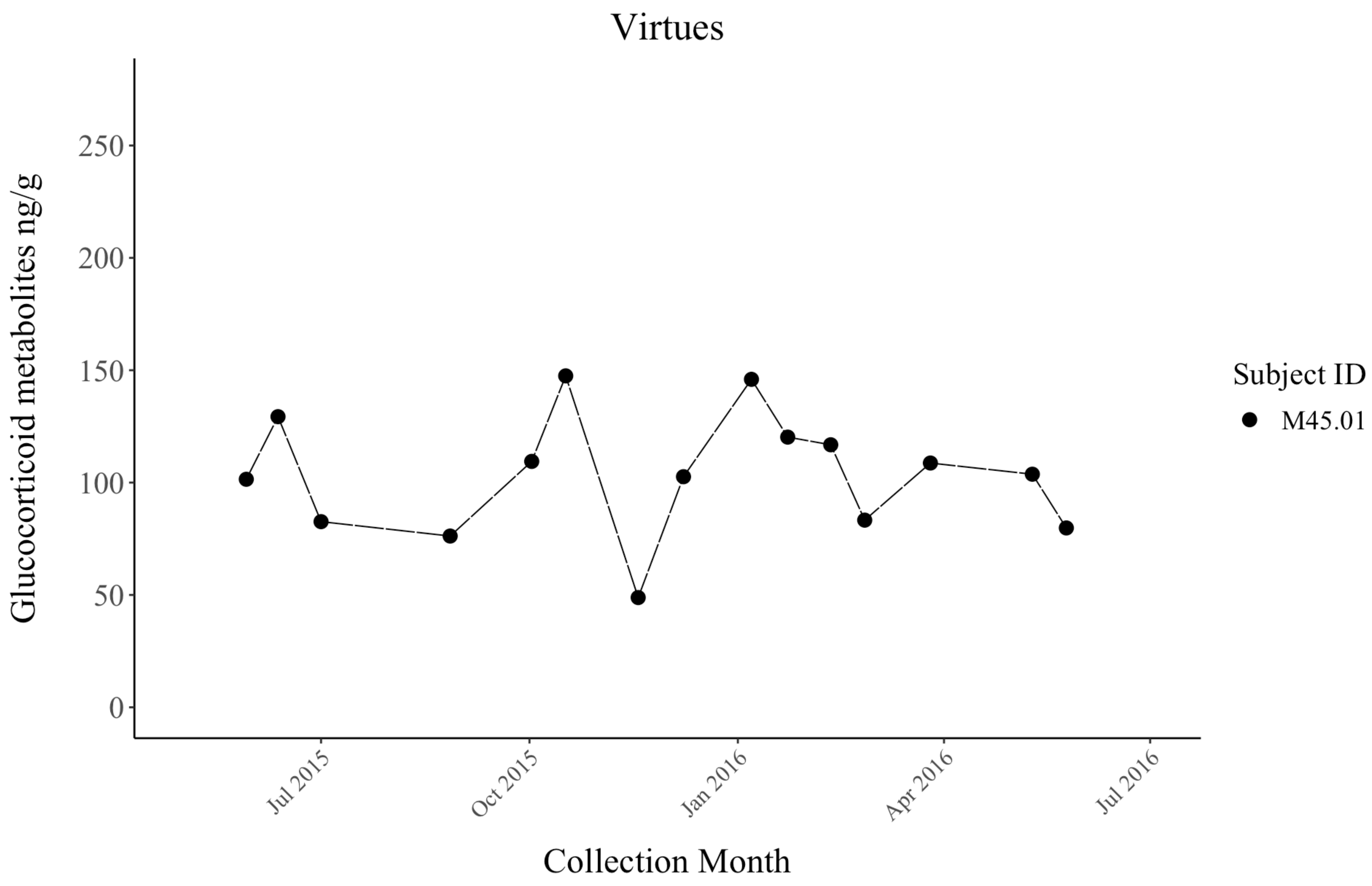

Virtues
Glucocorticoid metabolites ng/g
0
50
100
150
200
250
Jul 2015
Oct 2015
Jan 2016
Apr 2016
Jul 2016
Collection Month
Subject ID
M45.01

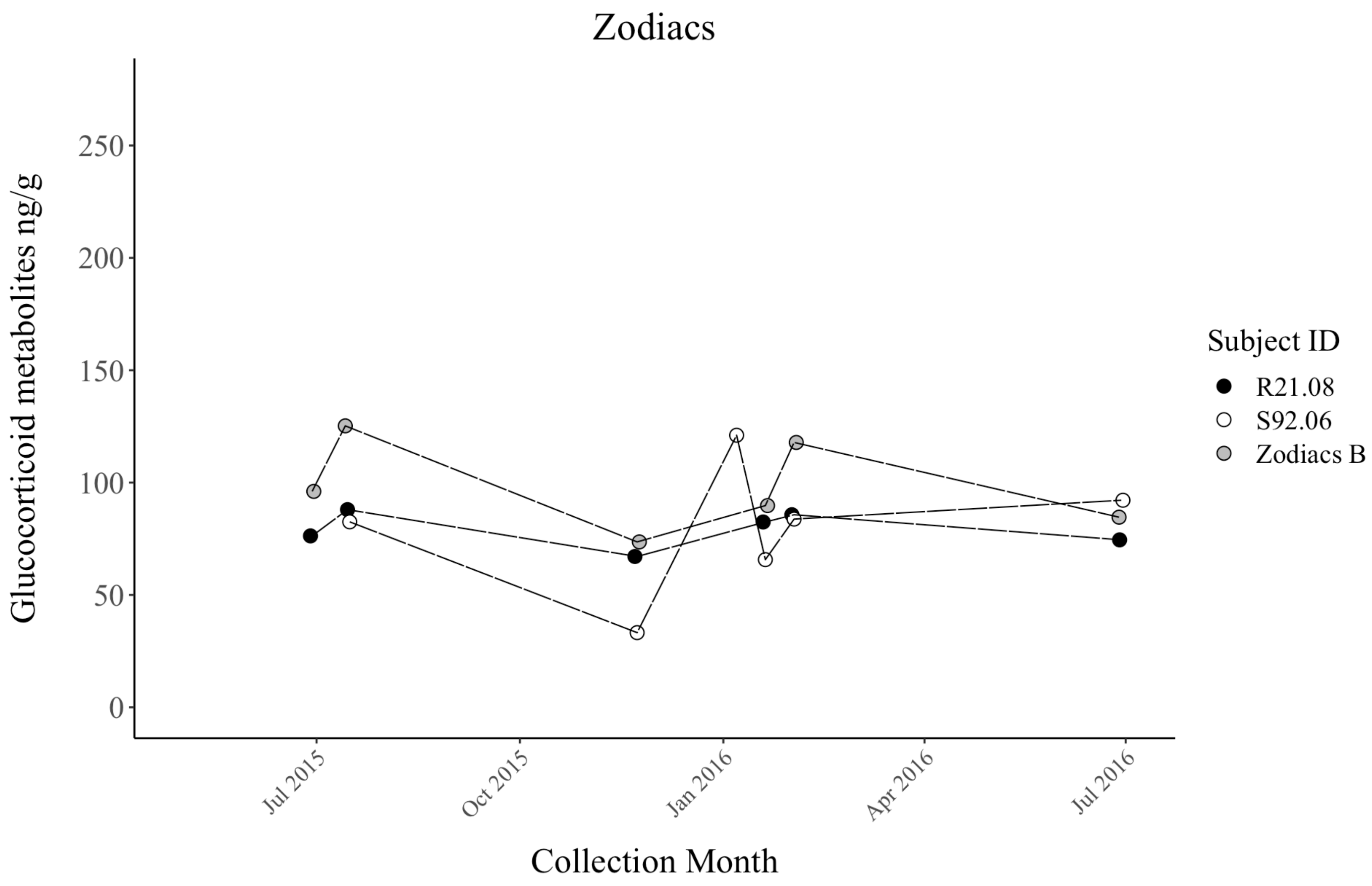

Zodiacs
Glucocorticoid metabolites ng/g
250
200
150
100
50
0
Jul 2015
Oct 2015
Jan 2016
Apr 2016
Jul 2016
Collection Month
Subject ID
R21.08
S92.06
Zodiacs B

Supplementary Figure 2: Time series graphs showing each study subject's fecal glucocorticoid metabolite concentration from one sample to the next, with subjects organized according to core group. Concentrations of a single individual oscillated according to season.

Supplementary Table 1: Results from the sample subset including strongylid fecal egg counts, with n = 446 dung samples from 37 elephants. The dotted line separates the first (top) and second (bottom) level of the model. The first level estimated individual-level intercepts, determining the correlation of unchanging variables with mean glucocorticoid concentrations. The second level used the estimated intercepts of the first level to determine the correlation of variables that changed with time according to when a sample was collected. Coefficients and covariates are ordered according to estimated effect size, from negative to positive, within each level and asterisks denote those for which the estimated 95% confidence interval did not overlap zero. NDVI stands for "normalized difference vegetation index," a measure of primary productivity.

Coefficient	Covariate	Estimate	95% CI lower	95% CI upper
b_5	with non-natal group	-0.34	-0.74	0.07
*b_2	*age mates	-0.25	-0.37	-0.13
*b_3	*number of samples	-0.12	-0.23	-0.01
b_1	adult caregivers	-0.09	-0.20	0.02
b_4	orphan status	0.07	-0.23	0.38
*γ_5	*mean NDVI	-0.85	-1.40	-0.30
γ_2	lactating	-0.11	-0.37	0.16
γ_1	age	-0.05	-0.23	0.13
γ_4	time of day	0.01	-0.08	0.10
γ_9	strongylid fecal egg counts	0.06	-0.04	0.16
γ_8	years without mom	0.08	-0.07	0.22
γ_6	pregnancy	0.08	-0.13	0.30
*γ_3	*time sample sat on ground	0.10	+0.00	0.19
*γ_7	*NDVI standard deviation	0.89	0.37	1.42

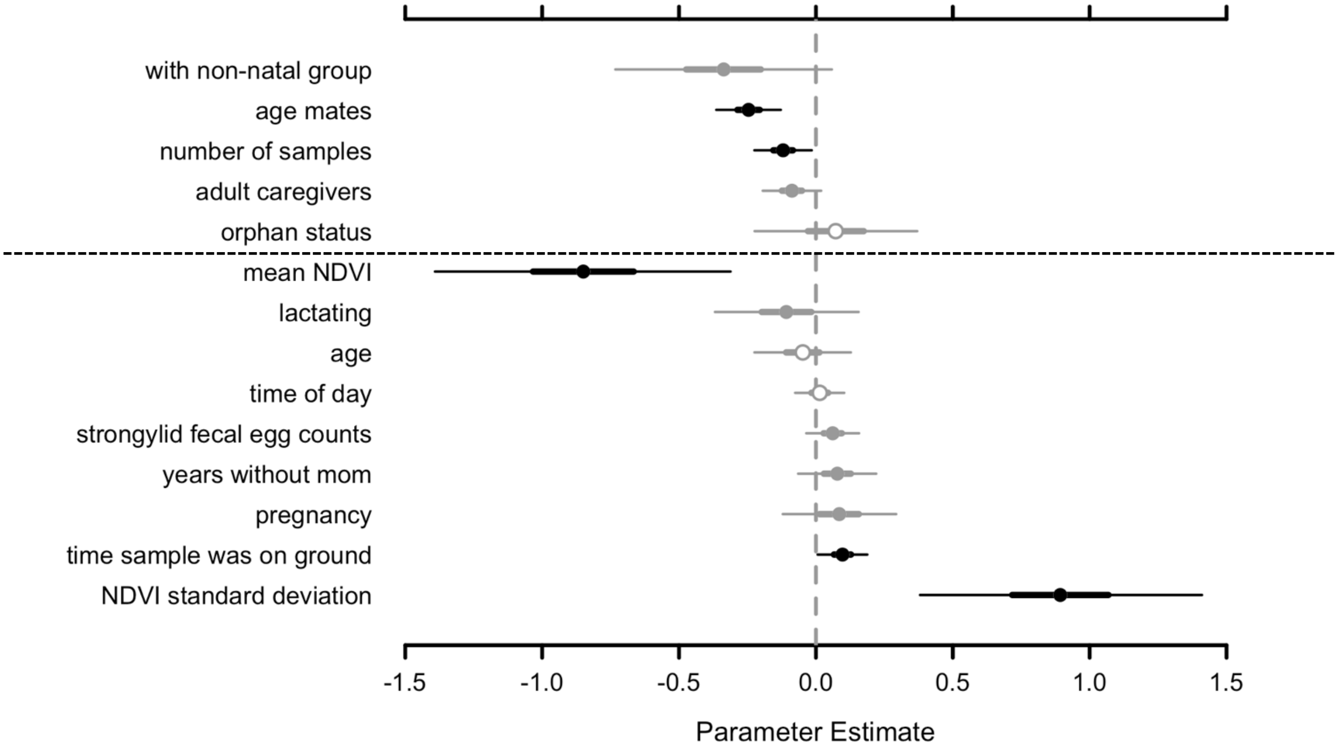

Supplementary Figure 3: Results from the sample subset including strongylid fecal egg counts, with n = 446 dung samples from 37 elephants. Black denotes estimates whose 95% confidence interval did not overlap zero, gray denotes estimates whose 50% confidence interval did not overlap zero, and open circles denote variables for which both confidence intervals overlap zero. The dotted line separates the first (top) and second (bottom) level of the model. The first level estimated individual-level intercepts, determining the correlation of unchanging variables with mean glucocorticoid concentrations. The second level used the estimated individual intercepts of the first level to determine the correlation of variables that changed with time according to when a sample was collected. Covariates are ordered according to estimated correlation size, from negative to positive, within each level. NDVI stands for “normalized difference vegetation index,” a measure of primary productivity.

SUPPLEMENTARY DISCUSSION

Previous studies have found stage of gestation correlates positively with fGCM concentrations in African elephants[1] and lactation correlates positively with circulating GC concentrations in captive Asian elephants (*Elephas maximus*)[2]. In our study, pregnancy was only slightly correlated with higher fGCM levels and not significantly, perhaps due to our course 0/1 categorization that did not incorporate stage of gestation. Moreover, lactating elephants had lower fGCM concentrations, albeit only slightly and not significantly. This may be because lactating females release more oxytocin, a hormone that inhibits adrenal activity and lowers GC secretion[3–5], which may have counterbalanced the effect of the energetic demands of lactation. (Oxytocin may further be the mechanism by which social buffering attenuates GC secretion; affiliative physical contact has been documented to release oxytocin in contexts unrelated to nursing and reproduction)[4,6].

We did not find that GC secretion increases with age in African elephants similar to Oduor *et al.* (2020)[7]. This may be because we did not sample from a wide age range, given elephants can live to be greater than 60 years old in the wild (Supplementary Figure 1). Differences between mature adults and young adults or calves may have been apparent if we had sampled from more coarsely separated age classes. Time of day showed an effect on fGCM concentrations in zoo elephants, with concentrations highest in the morning and lowest around midnight [8]. We did not sample across a large time range (Supplementary Figure 1C), and this could have obscured a similar effect in our study system. Zoo elephants may also have different diurnal rhythms than wild elephants after adjusting to human-driven schedules.

We suspected the resolution provided by fine-scale information on strongylid fecal egg counts from the same dung boluses sampled for glucocorticoids would unveil a positive

correlation with nematode parasite infection. However, we did not find support for a significant correlation of fGCM concentrations with strongylid FECs, agreeing with literature suggesting nematode parasites more rarely correlate with GCs than other types of parasites[9]. Interestingly, the effect of being with a non-natal core group was weaker in the model including FECs than in the model including all samples with no FECs. This may have simply been due to a lower overall sample size, but Parker *et al.* (2020) found that non-natal orphans have lower FECs[10], therefore including FECs might have drawn from variation due to non-natality if some of that variation was associated with lower strongylid infection.

Finally, years an orphan spent without her mother did not significantly correlate with fGCM concentrations, with a slightly positive estimated effect. As discussed in the main text, we would likely have to sample from orphans a shorter time after their mother's death to understand whether there are initial increases and how that may change over time.

Supplemental References

1. Foley, C. A. H., Papageorge, S. & Wasser, S. K. Noninvasive stress and reproductive measures of social and ecological pressures in free-ranging African elephants. *Conserv. Biol.* **15**, 1134–1142 (2001).
2. Glaeser, S. S., Edwards, K. L., Wielebnowski, N. & Brown, J. L. Effects of physiological changes and social life events on adrenal glucocorticoid activity in female zoo-housed Asian elephants (Elephas maximus). *PLoS One* **15**, 1–36 (2020).
3. DeVries, A. C. Interaction among social environment, the hypothalamic-pituitary-adrenal axis, and behavior. *Horm. Behav.* **41**, 405–413 (2002).
4. DeVries, A. C., Glasper, E. R. & Detillion, C. E. Social modulation of stress responses. *Physiol. Behav.* **79**, 399–407 (2003).

5. Reeder, D. A. M. & Kramer, K. M. Stress in free-ranging mammals: Integrating physiology, ecology, and natural history. *J. Mammal.* **86**, 225–235 (2005).

6. Wu, A. Social buffering of stress – Physiological and ethological perspectives. *Appl. Anim. Behav. Sci.* **239**, 105325 (2021).

7. Oduor, S. *et al.* Differing physiological and behavioral responses to anthropogenic factors between resident and non-resident African elephants at Mpala Ranch, Laikipia County, Kenya. *PeerJ* **8**, (2020).

8. Brown, J. L., Kersey, D. C., Freeman, E. W. & Wagener, T. Assessment of diurnal urinary cortisol excretion in Asian and African elephants using different endocrine methods. *Zoo Biol.* **29**, 274–283 (2010).

9. O'Dwyer, K., Dargent, F., Forbes, M. R. & Koprivnikar, J. Parasite infection leads to widespread glucocorticoid hormone increases in vertebrate hosts: A meta-analysis. *J. Anim. Ecol.* **89**, 519–529 (2020).

10. Parker, J. M., Goldenberg, S. Z., Letitiya, D. & Wittemyer, G. Strongylid infection varies with age, sex, movement and social factors in wild African elephants. *Parasitology* **147**, 348–359 (2020).
