# Supplementary material for: Social support correlates with glucocorticoid concentrations in wild African elephant orphans
Source: Commun Biol. 2022 Jul 14;5:630. doi: 10.1038/s42003-022-03574-8 (PMC9283395; doi:10.1038/s42003-022-03574-8)
Supplement: Supplementary file 3 — Reporting summary [file 42003_2022_3574_MOESM3_ESM.pdf]

nature portfolio

Corresponding author(s): Jenna Parker

Last updated by author(s): May 11, 2022

Reporting Summary

Nature Portfolio wishes to improve the reproducibility of the work that we publish. This form provides structure for consistency and transparency in reporting. For further information on Nature Portfolio policies, see our Editorial Policies and the Editorial Policy Checklist.

Statistics

For all statistical analyses, confirm that the following items are present in the figure legend, table legend, main text, or Methods section.

n/a	Confirmed	
☐	☒	The exact sample size (*n*) for each experimental group/condition, given as a discrete number and unit of measurement
☐	☒	A statement on whether measurements were taken from distinct samples or whether the same sample was measured repeatedly
☐	☒	The statistical test(s) used AND whether they are one- or two-sided<br>*Only common tests should be described solely by name; describe more complex techniques in the Methods section.*
☐	☒	A description of all covariates tested
☐	☒	A description of any assumptions or corrections, such as tests of normality and adjustment for multiple comparisons
☐	☒	A full description of the statistical parameters including central tendency (e.g. means) or other basic estimates (e.g. regression coefficient) AND variation (e.g. standard deviation) or associated estimates of uncertainty (e.g. confidence intervals)
☒	☐	For null hypothesis testing, the test statistic (e.g. *F*, *t*, *r*) with confidence intervals, effect sizes, degrees of freedom and *P* value noted<br>*Give P values as exact values whenever suitable.*
☐	☒	For Bayesian analysis, information on the choice of priors and Markov chain Monte Carlo settings
☐	☒	For hierarchical and complex designs, identification of the appropriate level for tests and full reporting of outcomes
☒	☐	Estimates of effect sizes (e.g. Cohen's *d*, Pearson's *r*), indicating how they were calculated

Our web collection on statistics for biologists contains articles on many of the points above.

Software and code

Policy information about availability of computer code

Data collection	Microsoft Excel was used to record data.
Data analysis	Analyses were run in RStudio version 1.1.463 with the package rjags version 4-10. The package bayesplot version 1.4.2 was used to visualize model fit. The packages ggplot2 and MCMCvis were used to make figures.

For manuscripts utilizing custom algorithms or software that are central to the research but not yet described in published literature, software must be made available to editors and reviewers. We strongly encourage code deposition in a community repository (e.g. GitHub). See the Nature Portfolio guidelines for submitting code & software for further information.

Data

Policy information about availability of data

All manuscripts must include a data availability statement. This statement should provide the following information, where applicable:

- Accession codes, unique identifiers, or web links for publicly available datasets
- A description of any restrictions on data availability
- For clinical datasets or third party data, please ensure that the statement adheres to our policy

We uploaded the fecal glucocorticoid concentration data to Harvard Dataverse and have supplied a link in the Data Availability statement. Due to the endangered conservation status of the African elephant, some of the other data are sensitive, therefore reviewers and readers should contact the authors if they would like to see those data.

Field-specific reporting

Please select the one below that is the best fit for your research. If you are not sure, read the appropriate sections before making your selection.

☐ Life sciences ☐ Behavioural & social sciences ☒ Ecological, evolutionary & environmental sciences

For a reference copy of the document with all sections, see nature.com/documents/nr-reporting-summary-flat.pdf

Ecological, evolutionary & environmental sciences study design

All studies must disclose on these points even when the disclosure is negative.

Study description	This study examined whether there are lasting differences in fecal glucocorticoid metabolite concentrations between wild orphan and nonorphan African elephants several years following the death of the orphans' mothers. Our overarching goal was to measure adrenal cortex activity in relation to maternal presence versus absence in a non-primate wildlife population and in the long-term. The study subjects came from a range of social contexts, from living in a relatively intact family to having joined an unrelated family because their family was so fractured. Therefore we also investigated the potential for the presence of other elephants to affect glucocorticoid secretion.
Research sample	This study was conducted on subjects chosen from a population of individually identified wild African elephants (Loxodonta africana) that have been monitored since 1998 in the Samburu and Buffalo Springs National Reserves of Kenya. We sampled from only female elephants because males disperse from their families anywhere from the ages of 5-18 and are therefore harder to find for reliable longitudinal sampling. We chose subjects so we had an adequate number of orphans matched with nonorphan controls (controls were the same age and part of the same group when possible), they came from a range of social contexts (i.e. from relatively intact families to families very fractured by poaching), they were often within the reserves so we could easily locate them for sampling, and based on whether they were a part of previous behavioral orphan studies to advance a wholistic understanding of orphaning's impact for elephants. We resulted with 37 subjects aged 7 - 21, n = 25 orphans and n = 12 nonorphans. Please see manuscript for more details.
Sampling strategy	Longitudinal sampling is important for comparing glucocorticoid concentrations among individuals because acute stressors can cause some samples to be atypical / misrepresentative, thus many samples are needed to accurately estimate an individual's typical baseline concentration. We therefore chose our sample size of individual elephants based on what was realistic for longitudinal dung sampling with a periodicity of 2 weeks (sometimes subjects were unreachable and more than 2 weeks passed between subsequent samples), deciding on 37 individuals. Our resultant sample size was 505 dung samples (of which 496 samples were analyzed, see below), with an average of 15 samples per individual. Please see manuscript for further details.
Data collection	We waited with the chosen elephant subjects until they produced a dung sample, collecting data associated with each sample in our notebooks (to be transferred to excel upon returning to camp) at the sampling event, and collecting the samples into bottles once the elephants had moved off. The samples were kept in a cool box until returning to the research camp, where they were stored in a freezer. Authors Jenna Parker and David Letitiya collected the majority of samples, with a few collected by author George Wittemyer and his family when Jenna and David were on leave.
Timing and spatial scale	We sampled dung over a period of 13 months from June 2015 to July 2016, with a minimum of two weeks between collecting samples from the same individual. We targeted two weeks to ensure adequate time to sample all individuals, and because more frequent sampling would have been too costly. However, we wanted to sample as frequently as necessary to capture seasonal effects and accumulate enough samples per individual for a realistic picture of her baseline glucocorticoid concentration. All samples were collected from within the Samburu and Buffalo Springs National Reserves of Kenya.
Data exclusions	Prior to analysis, we established a method to exclude samples that were likely misrepresentative, either due to acute stressors or measurement error. This method was to discard samples that were greater than 3 standard deviations from the mean fecal glucocorticoid metabolite concentration of the individual from which they were collected. This resulted in discarding 9 of the 505 samples, leaving 496 samples for analysis.
Reproducibility	This was a field study involving wildlife subjects, therefore repeating it under the exact same conditions was not technically or economically feasible.
Randomization	Randomization was not relevant to our study because we could not allocate elephants to specific groups.
Blinding	Blinding was not relevant to this study in the classical sense. However, the authors who analyzed the samples in the laboratory did not know the elephant subjects. Whereas the authors who knew the elephants might have had bias as to what a given individual's glucocorticoid concentration "should" be, the laboratory authors had no such bias because the elephant codes on the sample bottles meant nothing to them.

Did the study involve field work? ☒ Yes ☐ No

Field work, collection and transport

Field conditions	The field site is semi-arid, with annual average rainfall of 350 mm during two wet seasons from April - May and November - December. Temperature averages around 32 degrees Celsius during the day and 16 degrees Celsius at night.

Location	Sampling was conducted in the Samburu and Buffalo Springs National Reserves of Kenya, at 0.3 - 0.8 degrees N and 37 - 38 degrees E. These are unfenced reserves separated by a semi-permanent river called the Ewaso Ngiro, encompassing 220 square kilometers. Elevation ranges from 800 to 1200 m.
Access & import/export	We waited in vehicles for a subject to defecate, remaining inside until she and other elephants had moved off before driving to the sample and getting out to collect it. Permits were obtained from Kenya Wildlife Service (KWS); the National Commission for Science, Technology and Innovation (NACOSTI) of Kenya; the National Environment Management Authority (NEMA) of Kenya; and CITES prior to export of samples to the US.
Disturbance	We sometimes had to drive offroad to safely collect samples, but we did so only when necessary and went slowly to minimize damage.

Reporting for specific materials, systems and methods

We require information from authors about some types of materials, experimental systems and methods used in many studies. Here, indicate whether each material, system or method listed is relevant to your study. If you are not sure if a list item applies to your research, read the appropriate section before selecting a response.

Materials & experimental systems

n/a	Involved in the study
☒	☐ Antibodies
☒	☐ Eukaryotic cell lines
☒	☐ Palaeontology and archaeology
☐	☒ Animals and other organisms
☒	☐ Human research participants
☒	☐ Clinical data
☒	☐ Dual use research of concern

Methods

n/a	Involved in the study
☒	☐ ChIP-seq
☒	☐ Flow cytometry
☒	☐ MRI-based neuroimaging

Animals and other organisms

Policy information about studies involving animals; ARRIVE guidelines recommended for reporting animal research

Laboratory animals	The study did not involve laboratory animals.
Wild animals	Wild African elephants were observed in the field. These elephants are part of a larger long-term monitoring project that has been run by Dr. George Wittemyer and the NGO called Save the Elephants since 1998. Over 1000 elephants have been individually identified and are continually followed by a long-term monitoring team (in vehicles).
Field-collected samples	Dung samples were kept in a freezer at approximately -10 degrees Celsius until export, when they were shipped on dry ice to a laboratory at the Smithsonian Institute in the United States, where they were subsequently kept in a freezer until analysis.
Ethics oversight	Some of the elephants have been GPS-collared, a process that is overseen by the Kenya Wildlife Service (KWS) and their veterinarians. All research involving the elephants is approved by KWS.

Note that full information on the approval of the study protocol must also be provided in the manuscript.
