# Supplementary material for: Social support correlates with glucocorticoid concentrations in wild African elephant orphans
Source: Commun Biol. 2022 Jul 14;5:630. doi: 10.1038/s42003-022-03574-8 (PMC9283395; doi:10.1038/s42003-022-03574-8)
Supplement: Supplementary file 4 — Image license to publish [file 42003_2022_3574_MOESM4_ESM.pdf]

Image Licence to Publish

SPRINGER NATURE

Journal:	Commjnications Biology	(the "Journal")
Manuscript Number (if applicable):	COMMSBIO-21-2905	
Image Title or Description (including Animation, Images, Figures, Footage, Tables):	Image 1 – One of the study orphans from the Americans family (R28.03), at age 13, crossing the Ewaso N'giro River with her calf and her sister's calf.<br><br>Image 2 – Two of the study orphans from the Artists 2 family (R25.03 and R25.9002), at ages 13 and 14, resting with their calves. R25.03 has a floppy left ear, and R25.9002 (since killed by gunfire due to human elephant conflict) had a floppy right ear. They were always together, so at least had one pair of righted ears between them.	(the "Image")
Licensor(s):	Jenna Marie Parker	(the "Licensor(s)")
Insert Fee (**only** if applicable):	No fee	(the "Fee")

To: Springer Nature Limited (the "Publisher")

1 In consideration of the Publisher evaluating the Image for publication (and publishing the Image in its sole discretion) and/or payment by the Publisher to the Licensor(s) of the Fee (if applicable), the Licensor(s) grants to the Publisher from the date of this Agreement a non-exclusive, irrevocable, world-wide licence to publish, display, use and store the Image for single usage within a main editorial product and all related editions, revisions, translations and derivatives for the full period of copyright including all periods of renewal, extension and revival of the copyright and in all media whether now known or hereafter devised (including without limitation in print, digital and electronic form). The Image may also be used for marketing and promotional material (including without limitation on third party social media sites) but at all times in relation to the same editorial context mentioned above. The Publisher shall be entitled to make minor modifications to the Image for the purposes of resizing or reformatting the Image in the editorial product.

2 The Licensor(s) acknowledges that nothing in this Agreement shall obligate the Publisher to use or exploit the Image licensed to it by the Licensor(s). In the event that the Publisher decides not to use the Image, the Licensor(s) shall have no claim for loss of opportunity to enhance the Licensor(s)' reputation or for any other reason whatsoever.

3 The Licensor(s) undertakes, warrants and represents that:
(a) the Licensor(s) has full right, power and authority to enter into this Agreement and grant the rights granted herein;
(b) the Licensor is the sole legal and beneficial owner of all the rights, title and interests in and to all of the intellectual property rights in the Image or, in the event that the Licensor is not the sole legal and beneficial owner, the Licensor has all the necessary permissions, consents, approvals, licences, and authorities to grant the rights pursuant to this Agreement;
(c) the Image is the original work of the Licensor(s) and not copied (in whole or part) to any extent or at all;
(d) in the event that the Image contains any intellectual property rights or other proprietary rights belonging to a third party, the Licensor(s) has obtained the prior written consent from any such third party allowing the Licensor(s) to grant the rights contained herein;
(e) in the event that the Image contains any recognisable likeness of any living person(s), location or institution, the Licensor(s) has obtained the prior written consent from such person(s) whose likeness is reproduced, such institution and/or the owner of such location (as applicable) allowing the Licensor(s) to grant the rights contained herein;
(f) nothing in the Image is unlawful, obscene, defamatory, libellous, plagiarised, abusive, malicious, threatening, false, misleading, offensive, discriminatory, harassing, racist, sexist, indecent, pornographic, violates any right of privacy or publicity or infringes any intellectual property rights or other proprietary rights belonging to the Publisher or any other third party;
(g) nothing in the Image infringes any duty of confidentiality which the Licensor(s) may owe or violates any contract by which the Licensor(s) is bound.

4 Where the Image is used in the front cover of any of the Publisher's publications, all intellectual property rights in the compilation of such cover, including the image but only as part of the compilation, shall be vested solely in the Publisher and the Licensor(s) is not entitled to make or authorise any other person to make any use of such front cover compilation without the prior written consent of the Publisher.

5 The Licensor(s) hereby waives or agrees not to assert (where such waiver is not possible at law) any and all moral rights they may now or in the future hold in connection with the Image.

6 The Licensor(s) shall cooperate fully with the Publisher in relation to any legal action that might arise from the publication of the Image and the Licensor(s) shall give the Publisher access (at its reasonable request) to any relevant accounts, documents and records within the power or control of the Licensor(s).

7 No person other than a party to this Agreement shall have any rights to enforce any term of this Agreement and no variation of this Agreement shall be effective unless it is in writing and signed by the parties.

8 The Publisher may sub-license or assign these rights to its affiliates (such as Springer Nature America, Inc. and Scientific American). This Agreement shall be binding upon and inure to the benefit of the successors in business, licensees and assigns of the Publisher.

9 This Agreement shall be governed by and construed in accordance with the laws of England and Wales. The parties irrevocably agree that the courts of England and Wales shall have exclusive jurisdiction to settle any dispute or claim that arises out of or in connection with this Agreement or its subject matter or formation.

Signed for and on behalf of the Licensor(s) [Handwritten signatures only]:

Jenna Parker

Print Name: Jenna Parker

Date: 5/11/22

Address: 336 Edwards St, Fort Collins, CO 80524

Does the Image contain material from third parties?

☐ Yes (if yes, please ensure copies of the grant of rights are submitted with this form as indicated in clause 3(b), 3(d) and 3(e))

☒ No

Office use only:

Springer Nature Limited.
Registered office: The Campus, 4 Crinan Street, London, N1 9XW, UK. Company number 785998.
